# Co-expression and clinical utility of AR-FL and AR splice variants AR-V3, AR-V7 and AR-V9 in prostate cancer

**DOI:** 10.1186/s40364-023-00481-w

**Published:** 2023-04-05

**Authors:** Neele Wüstmann, Konstantin Egon Seifert, Verena Humberg, Julia Vieler, Norbert Grundmann, Julie Steinestel, Dorothee Tiedje, Stefan Duensing, Laura-Maria Krabbe, Martin Bögemann, Andres Jan Schrader, Christof Bernemann, Katrin Schlack

**Affiliations:** 1 Department of Urology, University Hospital Muenster, Albert-Schweitzer Campus 1 A1, 48149 Muenster, Germany; 2 Institute for Bioinformatics, University Hospital Muenster, Muenster, Germany; 3 Department of Urology, University Hospital Augsburg, Augsburg, Germany; 4 Molecular Urooncology, Department of Urology, University of Heidelberg School of Medicine, Heidelberg, Germany

**Keywords:** Androgen receptor, Androgen receptor splice variants, Androgen receptor targeted agents, Biomarker, Circulating tumor cells, Liquid biopsy, Metastatic castration resistant prostate cancer, Predictive, Prognostic

## Abstract

**Background:**

Androgen receptor (AR) splice variants (AR-Vs) have been discussed as a biomarker in prostate cancer (PC). However, some reports question the predictive property of AR-Vs. From a mechanistic perspective, the connection between AR full length (AR-FL) and AR-Vs is not fully understood. Here, we aimed to investigate the dependence of AR-FL and AR-V expression levels on AR gene activity. Additionally, we intended to comprehensively analyze presence of AR-FL and three clinically relevant AR-Vs (AR-V3, AR-V7 and AR-V9) in different stages of disease, especially with respect to clinical utility in PC patients undergoing AR targeted agent (ARTA) treatment.

**Methods:**

AR-FL and AR-V levels were analyzed in PC and non-PC cell lines upon artificial increase of AR pre-mRNA using either drug treatment or AR gene activation. Furthermore, expression of AR-FL and AR-Vs was determined in PC specimen at distinct stages of disease (primary (*n* = 10) and metastatic tissues (*n* = 20), liquid biopsy samples (*n* = 422), mCRPC liquid biopsy samples of *n* = 96 patients starting novel treatment). Finally, baseline AR-FL and AR-V status was correlated with clinical outcome in a defined cohort of *n* = 65 mCRPC patients undergoing ARTA treatment.

**Results:**

We revealed rising levels of AR-FL accompanied with appearance and increase of AR-Vs in dependence of elevated AR pre-mRNA levels. We also noticed increase in AR-FL and AR-V levels throughout disease progression. AR-V expression was always associated with high AR-FL levels without any sample being solely AR-V positive. In patients undergoing ARTA treatment, AR-FL did show prognostic, yet not predictive validity. Additionally, we observed a substantial clinical response to ARTA treatment even in AR-V positive patients. Accordingly, multivariate analysis did not demonstrate independent significance of AR-Vs in neither predictive nor prognostic clinical utility.

**Conclusion:**

We demonstrate a correlation between AR-FL and AR-V expression during PC progression; with AR-V expression being a side-effect of elevated AR pre-mRNA levels. Clinically, AR-V positivity relies on high levels of AR-FL, making cells still vulnerable to ARTA treatment, as demonstrated by AR-FL and AR-V positive patients responding to ARTA treatment. Thus, AR-FL and AR-V might be considered as a prognostic, yet not predictive biomarker in mCRPC patients.

**Supplementary Information:**

The online version contains supplementary material available at 10.1186/s40364-023-00481-w.

## Background

The full-length androgen receptor (AR-FL) is a driver of prostate cancer (PC) by acting as a transcription factor, thereby facilitating disease progression [1]. Development of novel AR targeting agents (ARTA), e.g., abiraterone and enzalutamide, has improved survival of metastatic castration resistant PC (mCRPC) patients [2–5]. Nonetheless, a substantial proportion of patients does not respond to treatment. Currently, there is no valid biomarker allowing stratification of patients who might benefit from these therapies. Thus, research on predictive biomarkers is urgently needed.

**Fig. 1:**
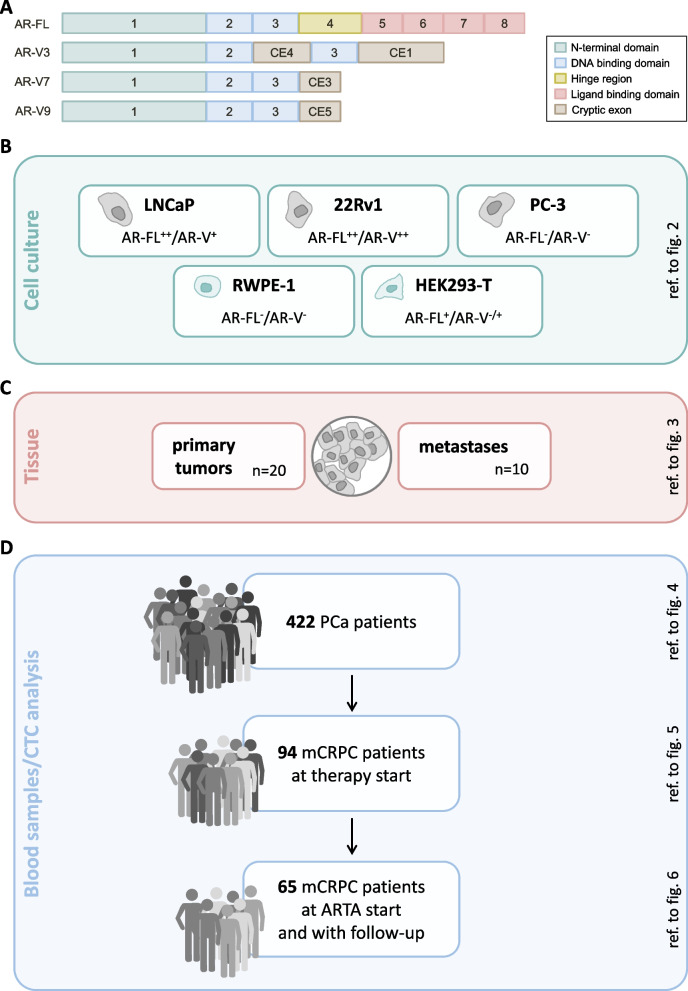
Structure of AR-FL and AR-Vs and experimental workflow. **A** Shown are the coding sequences of AR-FL (exon 1–8) as well as AR-V 3, 7 and 9. **B** Overview of experimental design. Analyses were performed in cell lines, primary and metastatic tumor tissues, and clinical CTC samples

AR splice variants (AR-Vs) have been discussed to predict non-response to ARTA [6]. These splice variant proteins lack a functional ligand binding domain (LBD), thus allowing them to act as transcription factors even in absence of ligands as well as presence of ARTAs (Fig. 1A). AR-V7, the most abundant AR-V, has gained clinical interest in prediction of non-response to ARTA. However, several reports describe substantial clinical response rates even in AR-V7 positive patients [7, 8]. Thus, its predictive property is still under debate [9–11]. Other AR-Vs, e.g., AR-V3 and AR-V9, have been found to be co-expressed in clinical PC specimen of all stages [12–18].

AR-FL and AR-Vs have been analyzed in a plethora of studies, describing appearance of AR-Vs as a main mechanism of ARTA resistance [19–23]. However, the exact processes, by which AR-V expression is induced or regulated, is poorly understood. Given a shared AR pre-mRNA origin, AR-V expression is likely dependent on AR gene regulation. Thus, we aimed to determine a connection between AR-FL and AR-Vs in clinical samples at distinct stages of disease. Furthermore, we intended to shed light on how AR-V expression might be regulated in correlation to AR-FL. Lastly – considering recent studies analyzing the clinical utility of AR-Vs as biomarker – we comprehensively analyzed the clinical value of both AR-FL and AR-Vs in liquid biopsy samples of mCRPC patients undergoing ARTA treatment.

## Methods

### Study design and ethics approval

We performed detection of AR-FL and AR-Vs (AR-V3, AR-V7 and AR-V9) (Fig. 1A) in a set of different human prostate (cancer) cell lines with different AR-FL and AR-V transcription levels (Fig. 1B): RWPE-1 cells (AR-FL^−^/AR-V^−^), LNCaP (AR-FL^++^/AR-V^+^), 22Rv1 (AR-FL^++^/AR-V^++^), PC-3 (AR-FL^−^/AR-V^−^). These cell lines represent different stages of prostate cancer (Figure S1): RWPE-1 cells (healthy prostate epithelium), LNCaP (hormone-sensitive stage), 22Rv1 (castration-resistant stage), PC-3 (neuroendocrine differentiated stage). For non-cancer HEK293-T cells, AR-FL and AR-V expression has been analyzed previously [24]. Given the non-prostate origin of these cells, we aimed to reanalyze AR-FL and AR-V expression and determined these cells being AR-FL + (low expression level) and variable in AR-V expression. Additionally, AR-FL and AR-V expression levels were analyzed in different clinical prostate cancer samples: primary PC tissue and lung and lymph node metastatic tissue samples as well as liquid biopsy CTC samples from mCRPC prostate cancer patients subdivided into the following groups: *n* = 422 PCa patients; *n* = 94 mCRPC patients prior new therapy; *n* = 65 patients starting ARTA treatment) (Fig. 1B). The local institutional review board approved the study and all patients provided written informed consent (2007–467-f-S). Primary PC tissue samples were obtained by the Department of Urology, Molecular Urooncology, University of Heidelberg School of Medicine, Heidelberg, Germany (votes 206/2005 and 207/2005 of the Ethics committee of the University of Heidelberg School of Medicine) and provided by the tissue bank of the National Center for Tumor Diseases (NCT) Heidelberg in accordance with the regulations of the tissue bank. RNA samples of metastases were obtained by the Prostate Cancer Biorepository Network (PCBN).

### Cell culture and treatment

Human cell lines were purchased from the Leibniz-Institute DSMZ GmbH (Braunschweig, Germany) (LNCaP, 22Rv1, PC-3 and HEK293-T) or ATCC® (RWPE-1) and cultured under matching protocols at 37 °C and 5% CO_2_. Media was purchased from SigmaAldrich (Pasching, Germany). Trypsin–EDTA, phosphate-buffered saline and fetal calf serum were purchased from Thermo Fisher Scientific (Waltham, MA, USA).

For AR modulation, we performed two different treatments: For ARTA related modulation, cells were cultured in the presence of 10 µM enzalutamide (Selleck Chemicals LLC, Houston, TX, USA) for up to 14 days. Induction of endogenous AR gene expression was performed using a modified CRISPR/dCas9 AR activation system ((#GA100263), Origene, Rockville, MD, USA). Cells in a 24 well plate were transfected with 500 ng gRNA or scramble control plasmids along with 150 ng enhancer plasmid using the ViaFect™ Transfection Reagent (Promega, Madison, WI, USA). RNA was isolated 48 h post transfection.

### mRNA isolation, cDNA synthesis and qPCR

For isolation of total RNA, we used the RNeasy® Mini kit (Qiagen, Hilden, Germany) following the manufactures guide. 500 ng of total RNA were reverse transcribed using the Primescript® Reverse Transcription Kit (Takara, Tokyo, Japan) or the Quantitect Reverse Transcription Kit along with gDNA wipeout buffer (Qiagen).

AR-FL expression was analyzed using TaqMan PCR assay (Hs00171172_m1) (Thermo Fisher Scientific). AR-V status was analyzed by using previously described custom-made TaqMan PCR assays specific for detection of AR-Vs AR-V3, AR-V7 and AR-V9 [17]. Assay sequences are listed in Table S1.

All qPCR runs were performed along with TaqMan PCR assays for housekeeping genes RPL37A (Hs01102345_m1) and HPRT1 (Hs99999909_m1) (Thermo Fisher Scientific). qPCR reactions were run using the Luna Mastermix on a QuantStudio 3 qPCR cycler (Thermo Fisher Scientific).

### CTC enrichment and determination

CTC analysis was conducted using a custom-made enrichment approach described in detail previously [25]. Briefly, blood samples were processed using the Dynabeads™ Epithelial Enrich Kit followed by mRNA isolation using the Dynabeads™ mRNA DIRECT™ Purification Kit (both Thermo Fisher Scientific). Contrary to cDNA synthesis of cell lines and tissue samples, cDNA synthesis of CTC samples was performed using the SuperScript™ IV VILO™ Master Mix (Thermo Fisher Scientific). For determination of CTCs we performed qPCR detection of KLK3-PSA mRNA using a KLK3-PSA TaqMan PCR assay (Hs03063374_m1) (Thermo Fisher Scientific). A patient sample was determined as CTC positive when displaying a qPCR signal for KLK3-PSA. CTC sample qPCR reactions were run using the TaqMan Fast Advanced Mastermix (Thermo Fisher Scientific).

### Copy number calculation

For copy number calculation, known number of oligonucleotides containing the exon spanning regions of AR-Vs – in decreasing concentrations – were used as template DNA in qPCR reactions (Fig. S2). Ct values were plotted against logarithmic levels of copy numbers. Using the linear equation, copy numbers of AR-FL and AR-Vs were calculated as copy numbers per 5 ml blood sample.

### Statistical analyses

The statistical assessment was performed using R software (version 4.1.3; R Foundation), SPSS-Statistics V25.0 (IBM Inc., Armonk, NY) and Prism 8 V8.4.3 (GraphPad Software, LLC., San Diego, CA). The descriptive statistics are reported as medians with interquartile ranges (IQR) or 95% confidence intervals (CI) for continuous variables and as frequencies and populations for categorical variables. Time-to-event outcomes (PFS and OS) were evaluated performing Kaplan–Meier analysis and Cox regression analysis for univariate and multivariate analyses.

## Results

### Expression of AR-Vs relies on increased levels of AR pre-mRNA

First, we thought to determine association of AR-FL and AR-V expression in vitro by using cell lines displaying different AR-FL and AR-V levels as well as diverse stages of prostate cancer (Fig. 1B, S1): RWPE-1 cells (healthy prostate epithelium; AR-FL^−^/AR-V^−^), LNCaP (hormone-sensitive stage; AR-FL^++^/AR-V^+^), 22Rv1 (castration-resistant stage; AR-FL^++^/AR-V^++^), PC-3 (neuroendocrine differentiated stage; AR-FL^−^/AR-V^−^) as well as non-prostate and non-cancer, yet AR positive HEK293-T (AR-FL^+^/AR-V^−/+^) cells. Cell lines were treated in two AR modulating conditions: enzalutamide treatment and endogenous AR gene induction using a modified CRISPR/dCas9 activation system.

**Fig. 2:**
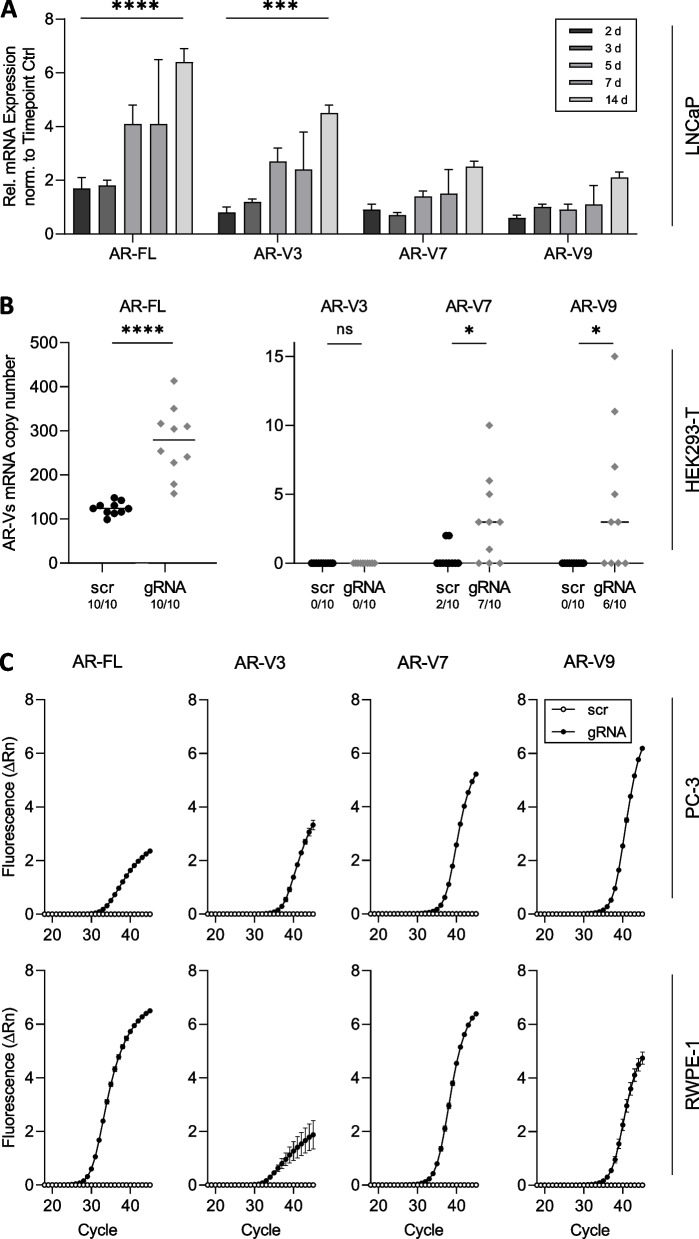
Elevated levels of endogenous AR induce appearance and increase of AR-Vs. **A** Fold change analysis (2 way ANOVA) of AR-FL and AR-Vs in LNCaP cells treated with enzalutamide for up to 14 days (*** represents *p* < 0.001, **** *p* < 0.0001). **B** Transcript copy number analysis of HEK293-T cells treated with CRISPR/dCas9 AR activation system. Shown are number of transcripts upon transfection with either scramble control plasmid (scr) or AR promotor specific guideRNA (gRNA). Numbers indicate number of positive samples within 10 transfections (ns represents *p* > 0.05, * *p* < 0.05, **** *p* < 0.0001). **C** Amplification curves of qPCR analyses of PC-3 and RWPE-1 cells treated with CRISPR/dCas9 AR activation system. Shown are amplification curves of AR-FL and AR-Vs. Cells were transfected with either scramble control plasmid (scr) or AR promotor specific guideRNA (gRNA)

LNCaP cells displayed significant increase in AR-FL mRNA levels within 14 days of treatment with enzalutamide (Fig. 2A). Also, we detected a significant increase of AR-V3 expression levels. AR-V7 and AR-V9 showed a slight increase. In enzalutamide-resistant 22Rv1 cells, no effect of enzalutamide on AR-FL and AR-Vs was observed (Fig. S3A). In HEK293-T cells, we robustly detected AR-FL mRNA, however without obvious effects of enzalutamide treatment. AR-Vs were randomly detected (Fig. S3B). In AR-FL and AR-V negative PC-3 and RWPE-1 cells, appearance of neither AR-FL nor AR-Vs was observed (data not shown).

When inducing endogenous AR gene expression using a modified CRISPR/dCas9 activation system, high levels of both endogenous AR-FL and AR-Vs could not be increased further in LNCaP and 22Rv1 cell lines (Fig. S3C). HEK293-T cells show low endogenous AR-FL expression and variable expression of AR-Vs without any treatment. When inducing AR, we detected a significant increase of AR-V7 and appearance of AR-V9. No AR-V3 mRNA was detected (Fig. 2B). In AR-FL/AR-V negative PC-3 and RWPE-1 cells, we detected appearance of both, AR-FL as well as AR-V mRNA upon AR gene induction with AR-FL displaying a higher expression level compared to AR-Vs (Fig. 2C).

### Appearance of AR-FL and AR-Vs in clinical samples at different stages of disease

**Fig. 3:**
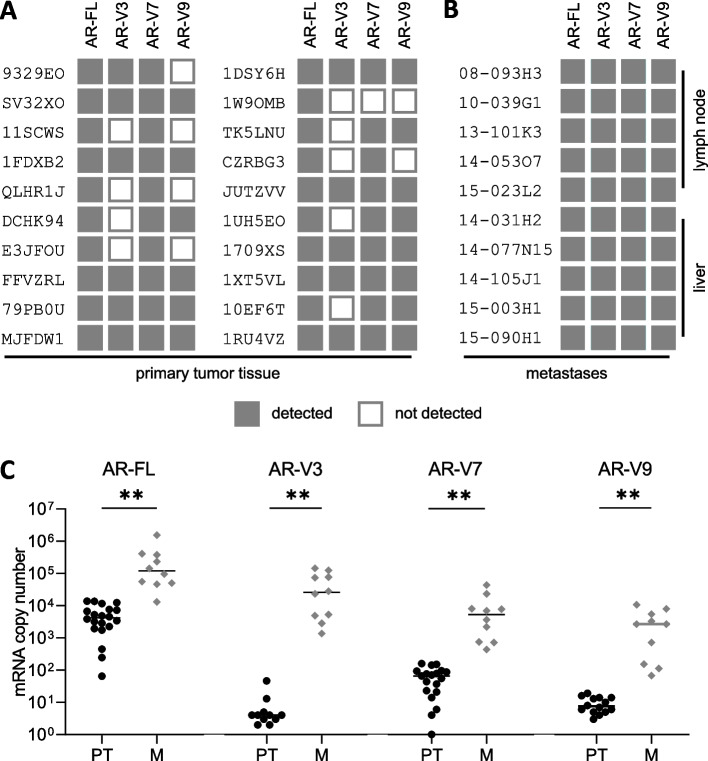
AR-FL and AR-Vs expression levels during disease progression. Shown are detection signals for AR-FL and AR-Vs in primary tumor tissue samples (**A**, *n* = 20) and metastatic tumor tissue samples (**B**, *n* = 10). **C** Copy number determination of AR-FL and AR-Vs in primary tumor tissue samples (PT) and metastatic tumor tissue samples (M) (** represents *p* < 0.01)

Next, we performed dichotomous detection analysis of AR-FL and AR-Vs, in primary PC tissue samples as well as metastatic biopsy samples (Fig. 3A, B). AR-FL was detected in all samples (100%). In primary PC samples, AR-V7 expression was most frequently detected (19/20 samples, 95%), whereas AR-V3 and AR-V9 were expressed in 11/20 (55%) and 14/20 (70%) samples, respectively (Fig. 3A). In metastatic tissue samples, we detected co-expression of all three AR-Vs in all samples (100%) (Fig. 3B).

Subsequently, we assessed copy numbers of AR-FL and AR-Vs in primary and metastatic tumor samples (Fig. 3C). Both AR-FL and AR-V expression levels were significantly lower in primary tumor tissue compared to metastatic tumor tissues.

**Fig. 4:**
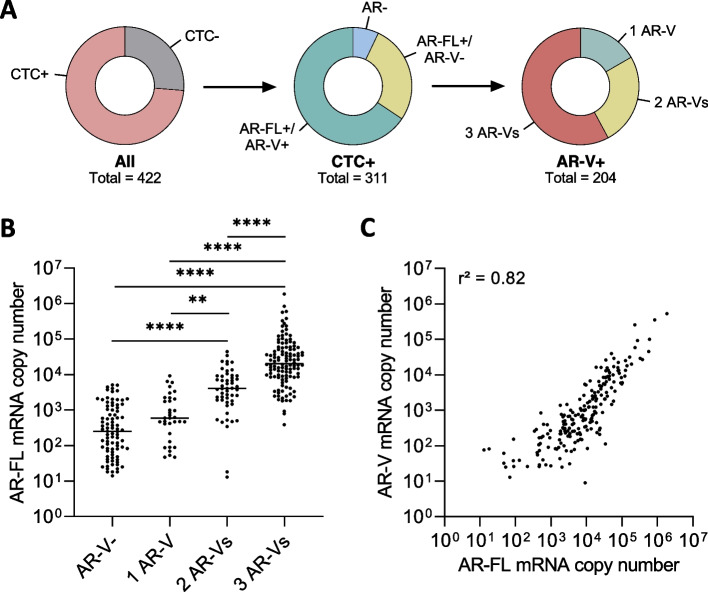
Comprehensive AR-FL and AR-V distribution analysis in prostate cancer patient CTC samples. **A** A cohort of *n* = 422 PC CTC samples was analyzed for presence of CTCs (left circle). Subsequently, the CTC + cohort (*n* = 311) was dichotomously analyzed for expression of AR-FL and AR-Vs (middle circle). Finally, the CTC + /AR-FL + /AR-V + cohort (*n* = 205) was analyzed for expression of one AR-V, two AR-Vs and three AR-Vs (right circle). **B** Analysis of AR-FL transcript copy numbers in CTC + /AR-FL + samples (*n* = 289) separated by number of AR-Vs detected (** represent < 0.01, **** < 0.0001). **C** Correlation analysis of AR-FL transcript copy numbers (x-axis) and AR-V transcript copy numbers (y-axis)

We then determined AR-FL and AR-V expression in circulating tumor cell (CTC) samples of *n* = 422 PC patients (Fig. 4A). One hundred and eleven patients (26.3%) did not display presence of CTCs, whereas CTCs were detected in 311 (73.7%) of the patients. Of those 311 CTC + patients, 22 did not show expression of any AR (7.1%). Eighty-five patients (27.3%) were found to be AR-FL + , without expression of AR-Vs. The remaining 204 (65.6) patients were found to be AR-FL + /AR-V + . Of those 204 AR-FL + /AR-V + patients, 34 (16.7%), 52 (25.5%) and 118 (57.8%) patients displayed expression of a single AR-V (CTC + /1 AR-V), two AR-Vs (CTC + /2 AR-Vs) and all three AR-Vs (CTC + /3 AR-Vs), respectively. Within the CTC + /AR-V + group (*n* = 204; 48.6% of total *n* = 422 samples), AR-V7 was found to be the most abundant AR-V (191 patients; 93.6%), whereas AR-V3 and AR-V9 were expressed in lower number of patients (159 patients (77.9%) and 142 patients (69.6%) respectively). These results demonstrate co-expression of AR-FL and AR-Vs in clinical liquid biopsy samples. Additionally, AR-V expression does not occur without simultaneous expression of AR-FL.

When analyzing expression levels of AR-FL, we detected the lowest AR-FL levels in samples displaying no AR-V expression compared to highest AR-FL levels in samples also displaying all three AR-Vs (Fig. 4B). We also noticed strong correlation of both AR-FL and AR-V expression levels with a slight shift to higher levels of AR-FL, suggesting the need of increased AR pre-mRNA transcripts ahead of AR-V appearance (Fig. 4C).

### Appearance of AR-FL and AR-Vs in mCRPC patients at distinct lines of treatment

**Fig. 5:**
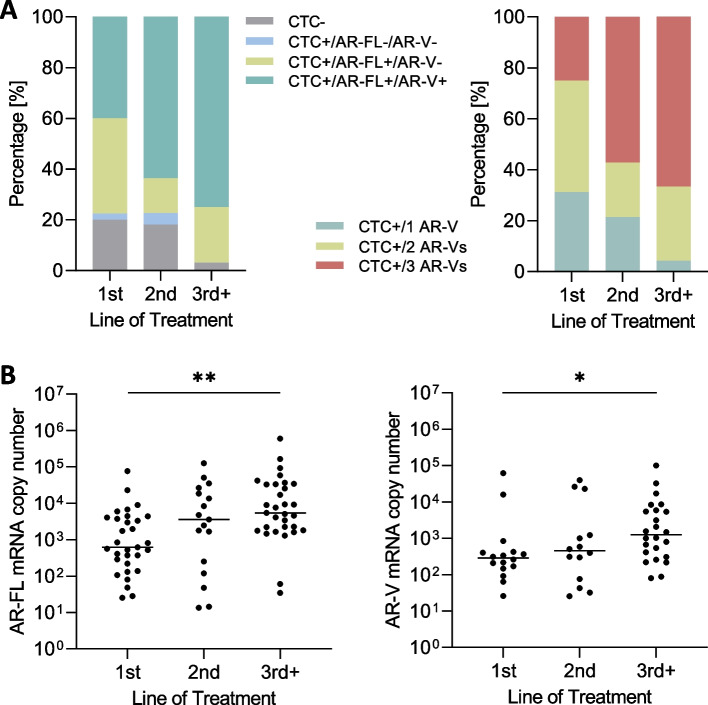
AR-FL and AR-V distribution and expression in *n* = 94 mCRPC patients. **A** Shown are the percentages of 4 different subgroups (CTC-, CTC + /AR-FL-/AR-V-, CTC + /AR-FL + /AR-V- and CTC + /AR-FL + /AR-V + ; left panel) as well as detailed distribution of AR-V numbers in CTC + /AR-FL + /AR-V + patients (right panel) at different lines of treatment. **B** Transcript copy number analyses of AR-FL copy numbers (left panel) and AR-V copy numbers (right panel) (* represents < 0.05, ** < 0.01)

Subsequently, we evaluated the distribution of all subgroups (CTC-, CTC + /AR-FL-/AR-V-, CTC + /AR-FL + /AR-V-, CTC + /AR-FL + /AR-V +) in a subgroup of *n* = 94 mCRPC patients starting either first line, second line or higher lines of treatment at the time of blood drawing. Treatment included both, ARTA and chemotherapeutic treatment. We detected 20.0% CTC- patients in first line treatment, 18.2% in second line and 3.1% in third or higher lines of treatment. 37.5% patients were identified being CTC + /AR-FL + /AR-V- in first line, 13.6% in second line and 21.9% in third or higher lines of treatment. 2.5%, 4.5% and 0.0% patients were identified to be CTC + /AR- in first, second and higher lines of treatment, respectively. For CTC + /AR-FL + /AR-V + expression, 40.0% of the patients were triple positive in first line, 63.7% in second line and 75.0% in third or higher lines of treatment (Fig. 5A left panel). When analyzing AR-V distribution in CTC + /AR-FL + /AR-V + patients, we detected appearance of one or two AR-Vs in 31.2% and 43.8% of the patients in first line, 21.4% and 21.4% in second line and 4.2% and 29.2% in third or higher lines of treatment, respectively. Three AR-Vs were detected in 25.0% of patients at first line, 57.2% in second line and 66.6% in third or higher lines of treatment (Fig. 5A right panel). Additionally, we noticed significantly higher expression levels of AR-FL along with higher levels of AR-V transcripts over different lines of treatment (Fig. 5B, *p* < 0.01 and *p* = 0.04). These results demonstrate higher appearance and expression levels of both AR-FL and AR-Vs in later stages of disease.

### AR-V expression and clinical outcome in a cohort of 65 patients undergoing ARTA

**Fig. 6:**
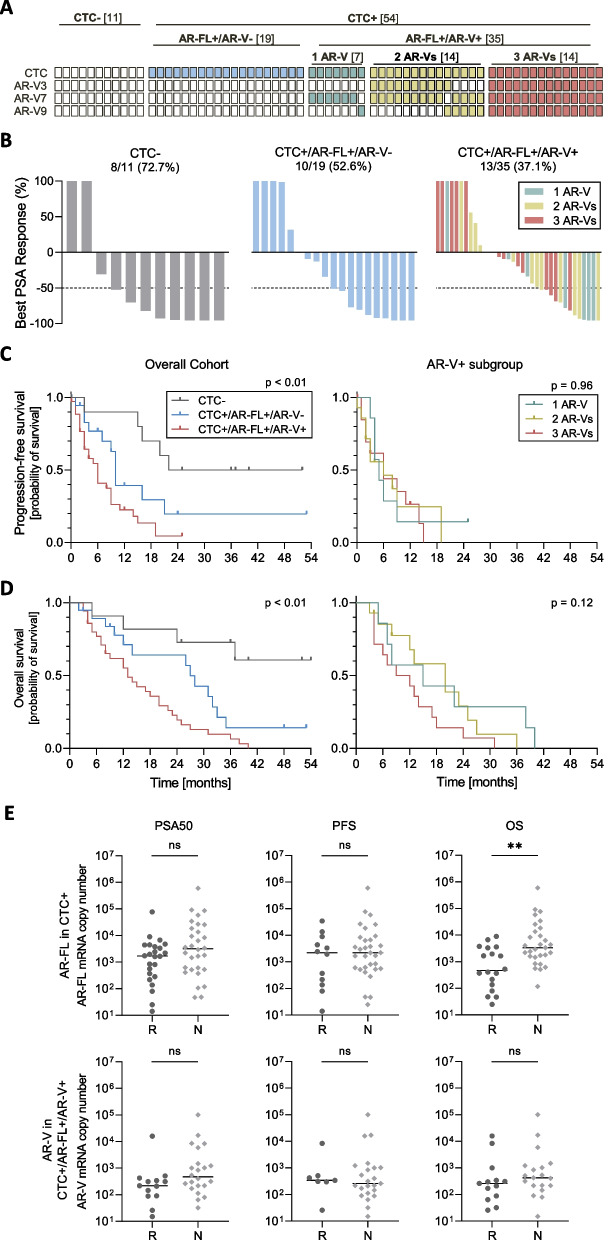
AR-V combination and clinical response in 65 mCRPC patients undergoing ARTA treatment. **A** Cohort overview. Shown are different subgroups according to CTC and AR-V status. **B** PSA response. Waterfall plots depicting best PSA responses in patients starting ARTA treatment according to CTC and AR-V status: CTC- (left panel), CTC + /AR-V- (middle panel), CTC + /AR-V + (right panel). The dotted line illustrates the threshold of PSA 50% decline defining a PSA response. Numbers indicate responding patients including percentages. **C**, **D** Kaplan–Meier curves indicating PFS (**C**) and OS (**D**) according to CTC/AR-FL/AR-V status (left panels) and numbers of AR-Vs in AR-V + patients (right panels). **E** Comparison of AR-FL and AR-Vs mRNA copy numbers per blood sample (5 ml) in AR-FL + (upper panels) and AR-V + patients (lower panels) regarding PSA response (left panels), PFS (middle panels) and OS (right panels) categorized into responders (R) and non-responders (N). P-values (Mann–Whitney test) are as follows: AR-FL in CTC + PSA50: *p* = 0.13, PFS: *p* = 0.49, OS: *p* < 0.01; AR-Vs in CTC + /AR-FL + /AR-V + PSA50: *p* = 0.07, PFS: *p* = 0.81, OS: *p* = 0.19

A subgroup of patients (*n* = 65 patients) was comprehensively analyzed for AR-V expression before starting ARTA treatment (abiraterone = 46, enzalutamide = 19). At the time of study closure in March 2021, median follow-up time was 14 (IQR 8–31) months. Baseline characteristics are presented in Table S2. Eleven patients (16.9%) were CTC-, 19 (29.2%) CTC + /AR-V- and 35 (54.0%) CTC + /AR-V + . All CTC + patients were also positive for expression of AR-FL. Among the group of AR-V + patients, seven (20.0%), 14 (40.0%) and 14 (40.0%) patients displayed expression of one AR-V, two AR-Vs or three AR-Vs, respectively. AR-V7 was the most abundant AR-V, detected in 33 (50.8%) patients followed by AR-V3 in 24 (36.9%) and AR-V9 in 20 (30.8%) patients (Fig. 6A). We observed a PSA response in 31 (47.7%) patients. CTC- patients showed a PSA response in eight (72.7%) cases, CTC + /AR-V- patients in ten (52.6%) and CTC + /AR-V + patients in 13 (37.1%) cases (Fig. 6B).

For the overall cohort, median PFS was 9 months (CI 7.1–10.9), within the three subgroups it was not reached for CTC- patients, 10 months (CI 8.9–11.1) for CTC + /AR-FL + /AR-V- patients and 6 months (CI 4.0–8.0) for CTC + /AR-FL + /AR-V + patients (Log-rank (Mantel-Cox) test, *p* < 0.01) (Fig. 6C left panel). Within the AR-V + subgroup, no significant differences (Log-rank (Mantel-Cox) test, *p* = 0.96) were observed between patients displaying expression of one (5 months (CI 2.4–7.6)), two (6 months (CI 0.0–13.5)) or even three AR-Vs (6 months (CI 1.2–10.8)) (Fig. 6C right panel).

Median OS for the overall cohort was 20 months (CI 10.5–29.5), within the three subgroups it was not reached for CTC- patients, 27 months (CI 23.5–30.5) for CTC + /AR-FL + /AR-V- patients and 13 months (CI 9.0–17.0) for CTC + /AR-FL + /AR-V + patients (Log-rank (Mantel-Cox) test, *p* < 0.01) (Fig. 6D left panel). When analyzing OS within the AR-V + subgroups, no significant differences (Log-rank (Mantel-Cox) test, *p* = 0.12) were detected among patients displaying expression of one (15 months (CI 0.0–33.0)), two (20 months (CI 9.5–30.5)) or even three AR-Vs (9 months (CI 0.0–18.2)) (Fig. 6D right panel).

Results of uni- and multivariate analysis for the different subgroups (overall cohort, CTC + /AR-FL + and CTC + /AR-FL + /AR-V +) are shown in Tables S3, S4, S5, S6, S7. Univariate analysis of the overall cohort showed that CTC positivity was significant for both PFS and OS. Within the CTC + /AR-FL + subgroup, AR-V positivity also showed significance in PFS and OS. Within the CTC + /AR-FL + /AR-V + group, no significant differences were observed, irrespective of number of AR-Vs. In multivariate analysis for the overall cohort, CTC positivity demonstrated significant differences in both, PFS and OS. In PFS, additionally Hb ≤ 12 at baseline showed significance. Furthermore, absence of a PSA decline ≥ 50% displayed significance in OS. Within the CTC + /AR-FL + cohort, Hb ≤ 12 at baseline was the only significant variable for OS.

### Quantification of AR-FL and AR-V transcript levels and correlation to PSA response, PFS and OS

Next, we determined AR-FL and AR-V transcript copy numbers in CTC + and AR-V + patients, respectively, and stratified patients into responding (R) and non-responding (N) subgroups. For AR-FL, we only noticed significantly higher copy numbers in patients demonstrating a worse OS. For none of the clinical outcome parameters, we detected significant differences of AR-V transcript copy numbers between responders (R) and non-responders (N) (Fig. 6E). These results suggest that actual expression levels of AR-Vs do not discriminate between responding and non-responding patients.

## Discussion

In this study, we aimed to analyze the connection of AR-FL and three supposedly clinically relevant AR-Vs, i.e., AR-V3, AR-V7 and AR-V9, in different stages of PC. Given controversial results on AR-Vs as biomarker, we also intended to comprehensively evaluate the predictive and prognostic power of AR-FL and AR-Vs in the context of ARTA treatment.

We demonstrate a correlation between both occurrence and expression levels of AR-FL and AR-Vs throughout the course of disease. AR-FL is a known driver in prostate cancer [1]. We detected apparent increase in AR-FL expression levels during disease progression. AR-Vs are known to be variably expressed at early stages of disease, while appearance increases when tumor progresses [12]. In line with this, we noticed rise in both incidence and AR-V copy numbers at later stages of disease. Interestingly, in case of clinical utility, we did not observe predictive power of neither AR-FL nor AR-Vs in patients undergoing ARTA treatment. The presented results are important from different perspectives.

From a biological perspective, the discovery of increasing mRNA levels of AR-Vs in PC has raised the question, whether these proteins might play a role in CRPC progression as well as ARTA treatment resistance [26–29]. This assumption is based on the unique structure of AR-Vs, lacking the LBD – the target structure of ARTA, yet being able to act as constitutively active transcription factors, eventually leading to activation of AR target genes [30]. Thus, it is tempting to speculate that patients exhibiting AR-Vs do not benefit from these treatment regimens. Given a shared pre-mRNA origin, AR-Vs are likely to be regulated by AR gene expression. Thus, high levels of AR pre-mRNA lead to variations in splicing processes, thereby facilitating the expression of AR-Vs. A mechanism for elevated AR pre-mRNA levels might be constant ARTA treatment as well as genomic AR amplification [26, 29, 31]. Elevated levels of AR pre-mRNA also lead to increasing levels of AR-FL. We now reveal increasing levels of AR-Vs in dependence of elevated levels of AR-FL in vitro as well as in patient samples at different stages of disease, thus strengthen the hypothesis that AR-Vs rely on a high level of AR pre-mRNA. Additionally, we were able to provide a direct link of elevated endogenous AR pre-mRNA levels leading to mRNA appearance of AR-Vs, even in the absence of ARTA treatment. This supports the premise of AR-Vs being a biological side-effect of increased AR gene activation.

From a clinical perspective, AR-Vs have been discussed as a resistance mechanism and thus, a tool for prediction of non-response to ARTA, mainly abiraterone and enzalutamide [19, 32, 33]. AR-V7 has been analyzed extensively in a plethora of distinct studies. Although initial results seemed promising, further reports questioned its predictive validity by demonstrating that patients did respond to ARTA despite expression of AR-V7 [7, 20]). Nonetheless, there still is rumor of whether AR-V7 might serve as a predictive biomarker in at least a subset of mCRPC patients, e.g., high risk patients [21, 34].

As with AR-FL, we noticed an increase in both actual number of AR-Vs and AR-V expression levels within higher lines of treatment. This is in line with a recent study demonstrating a correlation between AR-V appearance and a more advanced stage of disease [10, 35]. Thus, high levels of AR-FL and subsequent appearance of AR-Vs might be considered as a prognostic biomarker of disease progression or late stage of disease. However, we observed a substantial clinical response even in patients positive for AR-Vs. Remarkably, we did not detect significant differences in PFS and OS within the subgroup of AR-V + patients, irrespective of actual number of AR-Vs, demonstrating that even a combinatorial expression pattern is not sufficient to classify patients for non-response to ARTA. Furthermore, for the first time, we also reveal that actual levels of AR-V mRNA are not appropriate to stratify patients into responding and non-responding patients by demonstrating similar AR-V copy numbers in both subgroups. Ultimately, even high expression levels of AR-V mRNA do not preclude from considerable clinical responses. Unexpectedly, we noticed no significant differences in AR-FL copy numbers in responding and non-responding patients with respect to PFS. Thus, mRNA detection and quantification of both AR-FL and AR-Vs does not predict response or non-response to ARTA treatment. Consequently, none of these AR isoforms fulfills the requirements for a reliable predictive biomarker. With respect to OS, we observed a worse clinical outcome in patients displaying high copy numbers of AR-FL, yet not AR-Vs. Given however, that all AR-FL + patients in this cohort of ARTA treated patients are also CTC + , we hypothesize, that worse clinical outcome in AR-FL + patients is mainly based on presence of CTCs. High levels of AR-FL in this group might be a surrogate of higher number of CTCs. Thus, we conclude that – although AR-FL and AR-Vs might be of prognostic clinical value – a stratification of CTC- and CTC + patients is more valid. This is in line with a previous report of our group demonstrating that CTC, rather than AR-V7 determination, might be useful in mCRPC patient surveillance [35]. Additionally, CTC determination has already been approved as a prognostic marker [36–38] and thus, been integrated in guidelines of clinical trials. Changes of CTC numbers have been demonstrated to be a surrogate of clinical outcome [39].

The presented results finally lead to a main clinical consequence: virtually all cells expressing AR-Vs also express high levels of AR-FL, which still implies tumor cell vulnerability to ARTA treatment. Accordingly, we hypothesize that AR inhibition still provokes a clinical benefit even in patients expressing AR-V mRNA, presumably due to inhibition of the AR-FL protein and thus, alteration of the canonical AR signaling cascade. The main reason for AR-V related resistance mechanism depends on its nuclear localization to act as transcription factor even in the presence of ARTA [23, 33]. However, high levels of AR-V mRNA do not necessarily predict translation into functional protein and – as such—non-response. Astonishingly, even in patients displaying high levels of AR-V mRNA we noticed clinical response similar to patients with low levels of AR-V mRNA. Consequently, detection approaches analyzing the presence of AR-V mRNA are at risk of false-positive consideration of patients not responding to an otherwise valuable treatment.

## Conclusions

Our results demonstrate the value of AR-FL and AR-V as a prognostic, yet not predictive biomarker in the setting of ARTA for mCRPC patients. Thus, we postulate to avoid AR-FL and AR-vs as sole predictors for response to treatment. We assume that AR-V mRNA expression does not have a major mechanistic role in tumor progression, but rather is a side effect of elevated levels of AR pre-mRNA. This is underlined by a strong correlation of increased levels of both AR-FL and AR-Vs at distinct stages of disease. Hence, AR-V expression – at least on the mRNA level – should be regarded as an epiphenomenon related to a more advanced stage of disease rather than a biological mechanism of non-response to ARTA.

## Supplementary Information

**Additional file 1:** **Figure S1.** Cell lines at different stages of prostate cancer. Schematic overview of the clinical course of prostate cancer from healthy epithelium (left) to end stage prostate cancer and representative, established prostate (cancer) cell lines used in this study. HSPC: hormone sensitive PC; CRPC: castration resistant PC; NEDPC: neuroendocrine differentiated PC.

**Additional file 2:** **Figure S2.** cDNA copy number determination. Left panels: Overview of dsDNA oligonucleotides covering the spanning regions of KLK3-PSA, AR-FL, AR-V3, AR-V7 and AR-V9 TaqMan qPCR assays.Dotted line displays region of KLK3-PSA and AR-FL assay (targeted sequence confidential); Arrows and line displays forward and reverse primers as well as hydrolysis probe. Right panels: Standard curves for cDNA copy number quantification. Linear equations were used to determine copy numbers per 5ml blood sample.

**Additional file 3:** **Figure S3.** Endogenous AR modulation using either enzalutamide treatment or a modified CRISPR/dCas9 AR activation system. A) Fold change analysis of AR-FL and AR-Vs in 22Rv1 cells treated with enzalutamide for up to 14 days. B) AR-FL and AR-V copy number determination in HEK293-T cells treated with enzalutamide for up to 14 days. C) Amplification curves of qPCR analyses of LNCaP (upper panel) and 22Rv1 (lower panel) cells treated with CRISPR/dCas9 AR activation system. Shown are amplification curves of AR-FL and AR-Vs. Cells were transfected with either scramble control plasmid (scr) or AR promotor specific guideRNA (gRNA).

**Additional file 4:** **Table S1. **AR splice variant TaqMan detection assays.

**Additional file 5:** **Table S2. **Baseline characteristics of *n* = 65 patients with mCRPC starting treatment with abiraterone or enzalutamide.

**Additional file 6:** **Table S3. **Univariate analyses of biomarkers for PFS and OS in 65 mCRPC-patients on abiraterone or enzalutamide therapy (overall cohort). **TableS4. **Univariate analyses of biomarkers for PFS and OS in 54 mCRPC-patients on abiraterone or enzalutamide therapy (CTC+/AR-FL+ cohort). **Table S5. **Univariate analyses of biomarkers for PFS and OS in 35 mCRPC-patients on abiraterone or enzalutamide therapy (CTC+/AR-FL+/AR-V+ cohort). **TableS6. **Multivariate analyses of biomarkers for PFS and OS in 65 mCRPC-patients on abiraterone or enzalutamide therapy (overall cohort). **Table S7. **Multivariate analyses of biomarkers for PFS and OS in 54mCRPC-patients on abiraterone or enzalutamide therapy (CTC+/AR-FL+ cohort).

## Data Availability

The datasets used and/or analyzed during the current study are available from the corresponding author on reasonable request.

## Abbreviations

AR-FL: Androgen receptor full length
ARTA: Androgen receptor targeting agents
AR-V: Androgen receptor splice variant
CI: Confidence intervals
CTC: Circulating tumor cell
IQR: Interquartile range
LBD: Ligand binding domain
mCRPC: Metastatic castration resistant prostate cancer
OS: Overall survival
PC: Prostate cancer
PFS: Progression free survival
PSA: Prostate specific antigen
qPCR: Quantitative real time PCR
