# Supplementary material for: Co-expression and clinical utility of AR-FL and AR splice variants AR-V3, AR-V7 and AR-V9 in prostate cancer
Source: Biomark Res. 2023 Apr 5;11:37. doi: 10.1186/s40364-023-00481-w (PMC10074820; doi:10.1186/s40364-023-00481-w)
Supplement: Supplementary file 1 — Additional file 1: Figure S1. Cell lines at different stages of prostate cancer. Schematic overview of the clinical course of prostate cancer from healthy epithelium (left) to end stage prostate cancer and representative, established prostate (cancer) cell lines used in this study. HSPC: hormone sensitive PC; CRPC: castration resistant PC; NEDPC: neuroendocrine differentiated PC. [file 40364_2023_481_MOESM1_ESM.pptx]

## Slide 1
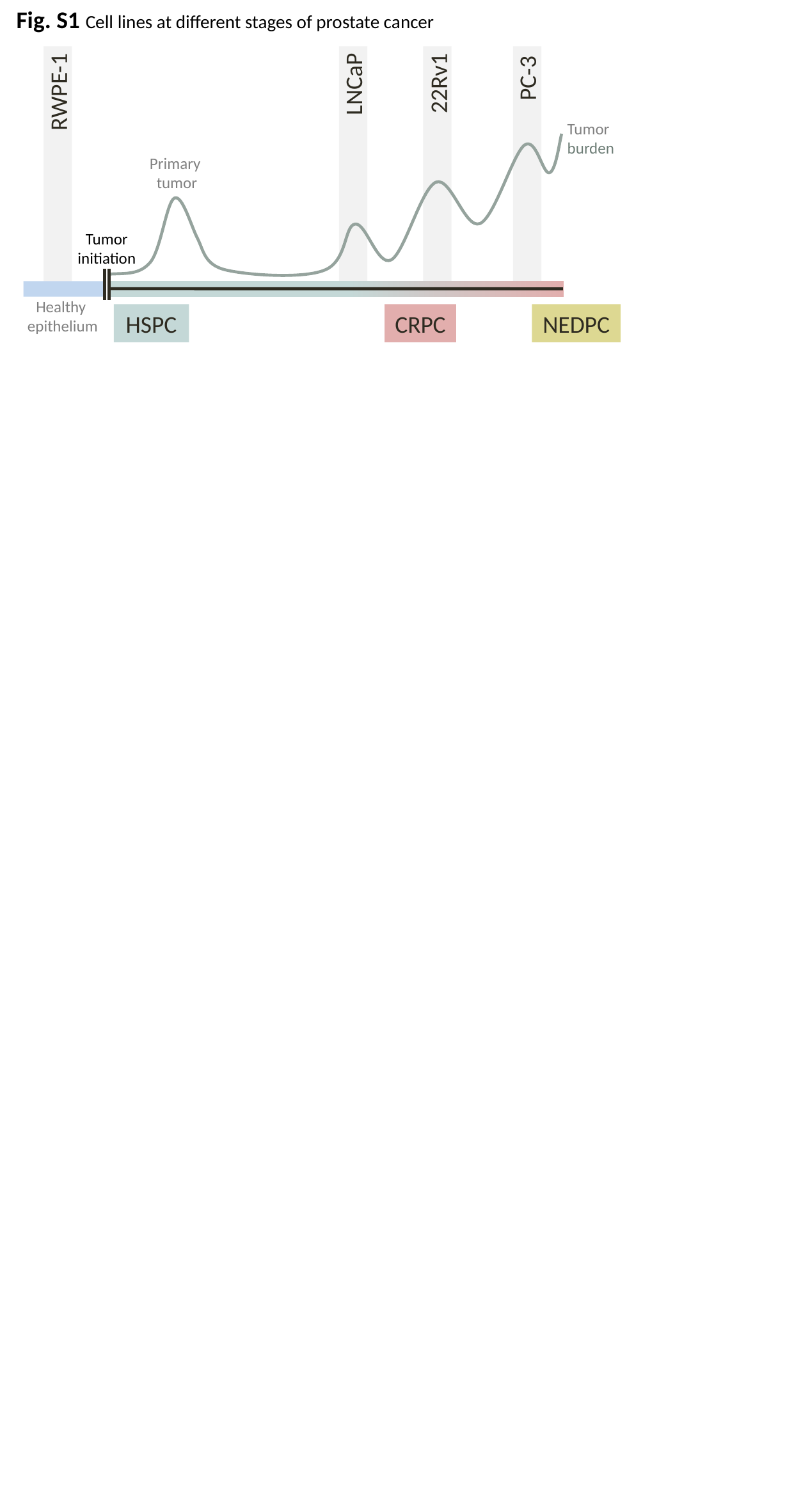

Fig. S1 Cell lines at different stages of prostate cancer
RWPE-1
PC-3
22Rv1
LNCaP
Tumor
burden
Primary
 tumor
Tumor initiation
Healthy
epithelium
HSPC
CRPC
NEDPC
