# Supplementary material for: Co-expression and clinical utility of AR-FL and AR splice variants AR-V3, AR-V7 and AR-V9 in prostate cancer
Source: Biomark Res. 2023 Apr 5;11:37. doi: 10.1186/s40364-023-00481-w (PMC10074820; doi:10.1186/s40364-023-00481-w)
Supplement: Supplementary file 2 — Additional file 2: Figure S2. cDNA copy number determination. Left panels: Overview of dsDNA oligonucleotides covering the spanning regions of KLK3-PSA, AR-FL, AR-V3, AR-V7 and AR-V9 TaqMan qPCR assays.Dotted line displays region of KLK3-PSA and AR-FL assay (targeted sequence confidential); Arrows and line displays forward and reverse primers as well as hydrolysis probe. Right panels: Standard curves for cDNA copy number quantification. Linear equations were used to determine copy numbers per 5ml blood sample. [file 40364_2023_481_MOESM2_ESM.pptx]

## Slide 1
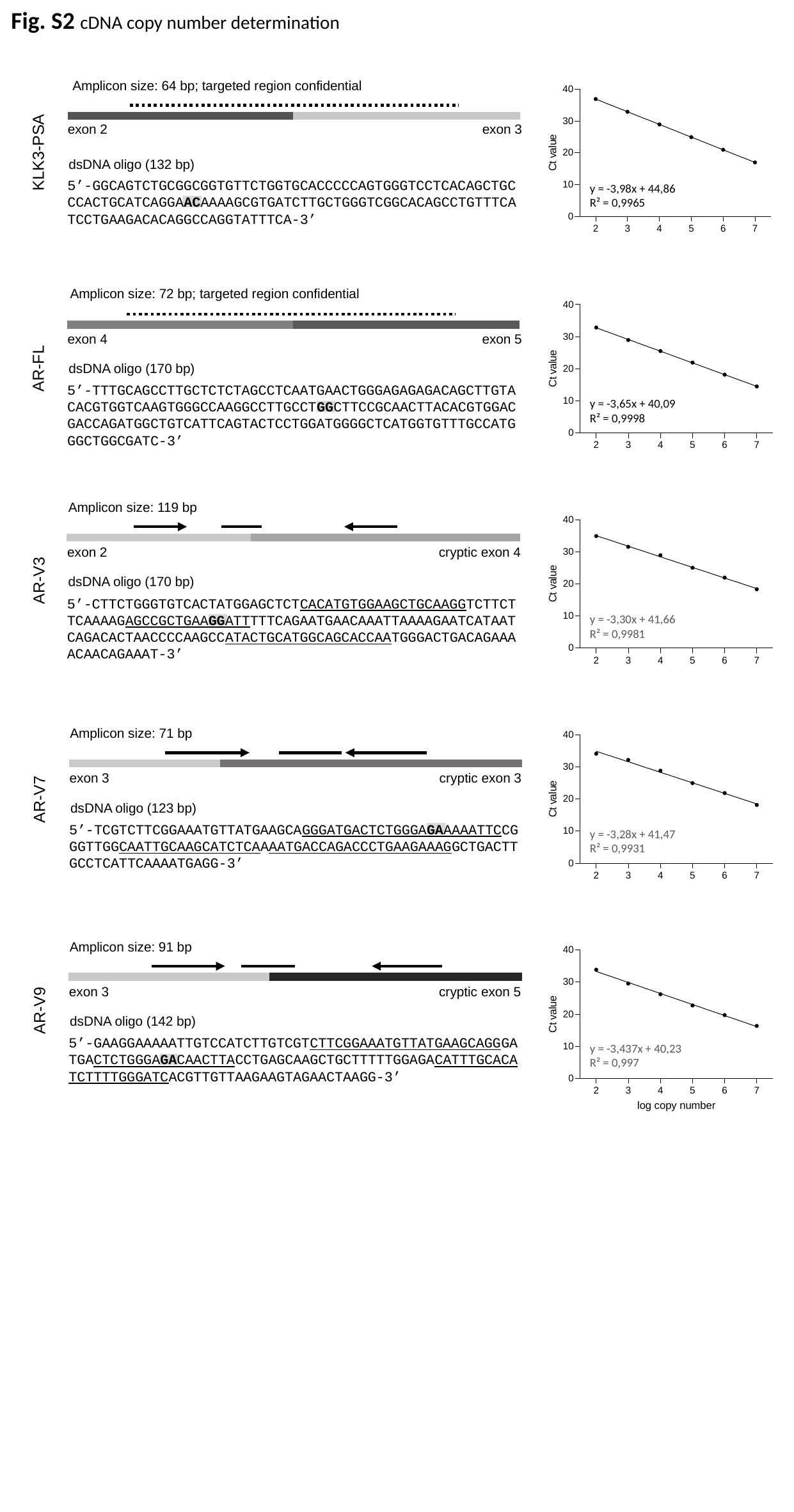

Fig. S2 cDNA copy number determination
y = -3,98x + 44,86
R² = 0,9965
y = -3,65x + 40,09
R² = 0,9998
y = -3,30x + 41,66
R² = 0,9981
y = -3,28x + 41,47
R² = 0,9931
y = -3,437x + 40,23
R² = 0,997
Amplicon size: 64 bp; targeted region confidential
exon 3
exon 2
dsDNA oligo (132 bp)
5’‑GGCAGTCTGCGGCGGTGTTCTGGTGCACCCCCAGTGGGTCCTCACAGCTGCCCACTGCATCAGGAACAAAAGCGTGATCTTGCTGGGTCGGCACAGCCTGTTTCATCCTGAAGACACAGGCCAGGTATTTCA‑3’
KLK3-PSA
Amplicon size: 72 bp; targeted region confidential
exon 5
exon 4
dsDNA oligo (170 bp)
5’‑TTTGCAGCCTTGCTCTCTAGCCTCAATGAACTGGGAGAGAGACAGCTTGTACACGTGGTCAAGTGGGCCAAGGCCTTGCCTGGCTTCCGCAACTTACACGTGGACGACCAGATGGCTGTCATTCAGTACTCCTGGATGGGGCTCATGGTGTTTGCCATGGGCTGGCGATC‑3’
AR-FL
Amplicon size: 119 bp
cryptic exon 4
exon 2
dsDNA oligo (170 bp)
5’‑CTTCTGGGTGTCACTATGGAGCTCTCACATGTGGAAGCTGCAAGGTCTTCTTCAAAAGAGCCGCTGAAGGATTTTTCAGAATGAACAAATTAAAAGAATCATAATCAGACACTAACCCCAAGCCATACTGCATGGCAGCACCAATGGGACTGACAGAAAACAACAGAAAT‑3’
AR-V3
Amplicon size: 71 bp
cryptic exon 3
exon 3
dsDNA oligo (123 bp)
5’‑TCGTCTTCGGAAATGTTATGAAGCAGGGATGACTCTGGGAGAAAAATTCCGGGTTGGCAATTGCAAGCATCTCAAAATGACCAGACCCTGAAGAAAGGCTGACTTGCCTCATTCAAAATGAGG‑3’
AR-V7
Amplicon size: 91 bp
cryptic exon 5
exon 3
dsDNA oligo (142 bp)
5’‑GAAGGAAAAATTGTCCATCTTGTCGTCTTCGGAAATGTTATGAAGCAGGGATGACTCTGGGAGACAACTTACCTGAGCAAGCTGCTTTTTGGAGACATTTGCACATCTTTTGGGATCACGTTGTTAAGAAGTAGAACTAAGG‑3’
AR-V9
