# Supplementary material for: Co-expression and clinical utility of AR-FL and AR splice variants AR-V3, AR-V7 and AR-V9 in prostate cancer
Source: Biomark Res. 2023 Apr 5;11:37. doi: 10.1186/s40364-023-00481-w (PMC10074820; doi:10.1186/s40364-023-00481-w)
Supplement: Supplementary file 3 — Additional file 3: Figure S3. Endogenous AR modulation using either enzalutamide treatment or a modified CRISPR/dCas9 AR activation system. A) Fold change analysis of AR-FL and AR-Vs in 22Rv1 cells treated with enzalutamide for up to 14 days. B) AR-FL and AR-V copy number determination in HEK293-T cells treated with enzalutamide for up to 14 days. C) Amplification curves of qPCR analyses of LNCaP (upper panel) and 22Rv1 (lower panel) cells treated with CRISPR/dCas9 AR activation system. Shown are amplification curves of AR-FL and AR-Vs. Cells were transfected with either scramble control plasmid (scr) or AR promotor specific guideRNA (gRNA). [file 40364_2023_481_MOESM3_ESM.pptx]

## Slide 1
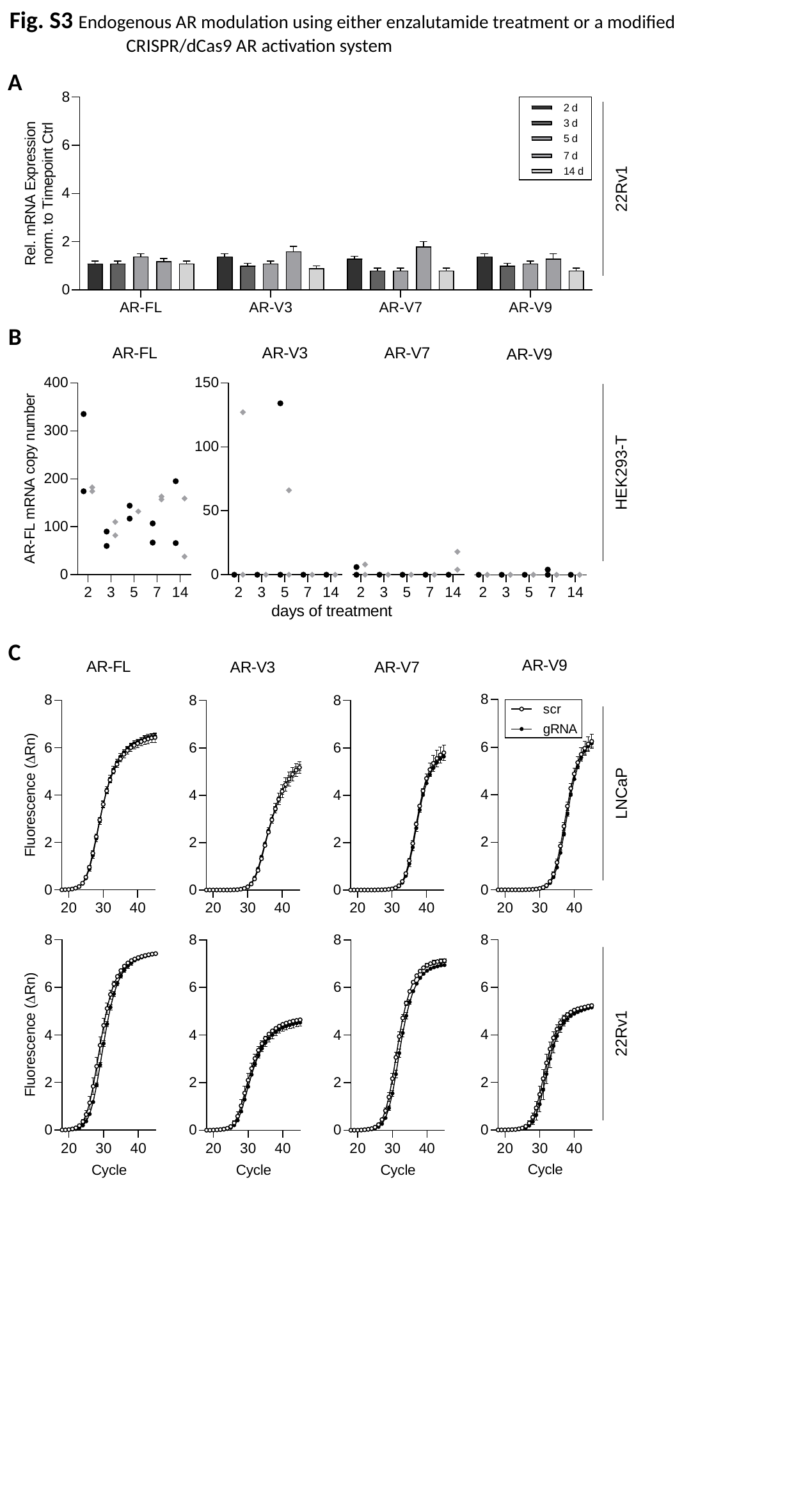

Fig. S3 Endogenous AR modulation using either enzalutamide treatment or a modified 			 CRISPR/dCas9 AR activation system
A
22Rv1
B
HEK293-T
C
LNCaP
22Rv1
