# Supplementary material for: Co-expression and clinical utility of AR-FL and AR splice variants AR-V3, AR-V7 and AR-V9 in prostate cancer
Source: Biomark Res. 2023 Apr 5;11:37. doi: 10.1186/s40364-023-00481-w (PMC10074820; doi:10.1186/s40364-023-00481-w)
Supplement: Supplementary file 5 — Additional file 5: Table S2. Baseline characteristics of n = 65 patients with mCRPC starting treatment with abiraterone or enzalutamide. [file 40364_2023_481_MOESM5_ESM.docx]

Table S2: Baseline characteristics of patients with mCRPC starting treatment with abiraterone or enzalutamide

|  | **Total** |
| --- | --- |
|  |  |
| **Patients [n]** | 65 |
| **Age, median [years] (IQR)** | 66 (62-76) |
| **Therapy [n] (%)**  **abiraterone**  **enzalutamide** | 46 (70.8)  19 (29.2) |
| **Prior abiraterone or enzalutamide [n] (%)** | 22 (33.9) |
| **Prior use of docetaxel [n] (%)** | 25 (38.5) |
| **Line of therapy [n] (%)**  **1^st^**  **2^nd^**  **3^rd^ or higher** | 33 (50.8)  15 (23.1)  17 (26.2) |
| **PSA doubling time < 3 months [n] (%)** | 28 (43.1) |
| **ECOG performance status (all) [n] (%)**  **0**  **≥ 1** | 53 (94.6)  3 (5.4) |
| **Gleason-Score ≥ 8 [n] (%)** | 47 (77.1) |
| **Median PSA at baseline [ng/ml] (IQR)** | 24.3 (9.3-114.0) |
| **Median LDH at baseline [U/l] (IQR)** | 223 (197-275) |
| **Median ALP at baseline [U/l] (IQR)** | 106 (76-169) |
| **Hb < 12 g/dl [n] (%)** | 21 (32.3) |
| **Elevated LDH at baseline [n] (%)** | 27 (41.5) |
| **Elevated ALP at baseline [n] (%)** | 21 (32.3) |
| **Bone protection [n] (%)** | 14 (21.5) |
| **Presence of lymph node metastases [n] (%)** | 37 (56.9) |
| **Presence of bone metastases [n] (%)** | 51 (78.5) |
| **Presence of visceral metastases [n] (%)** | 6 (9.2) |

Abbreviations: ALP: alkaline phosphatase; ECOG: eastern co-operative oncology group; Hb: hemoglobin; IQR: interquartile range; LDH: lactate dehydrogenase; PSA: prostate specific antigen
