# Supplementary material for: Co-expression and clinical utility of AR-FL and AR splice variants AR-V3, AR-V7 and AR-V9 in prostate cancer
Source: Biomark Res. 2023 Apr 5;11:37. doi: 10.1186/s40364-023-00481-w (PMC10074820; doi:10.1186/s40364-023-00481-w)
Supplement: Supplementary file 6 — Additional file 6: Table S3. Univariate analyses of biomarkers for PFS and OS in 65 mCRPC-patients on abiraterone or enzalutamide therapy (overall cohort). TableS4. Univariate analyses of biomarkers for PFS and OS in 54 mCRPC-patients on abiraterone or enzalutamide therapy (CTC+/AR-FL+ cohort). Table S5. Univariate analyses of biomarkers for PFS and OS in 35 mCRPC-patients on abiraterone or enzalutamide therapy (CTC+/AR-FL+/AR-V+ cohort). TableS6. Multivariate analyses of biomarkers for PFS and OS in 65 mCRPC-patients on abiraterone or enzalutamide therapy (overall cohort). Table S7. Multivariate analyses of biomarkers for PFS and OS in 54mCRPC-patients on abiraterone or enzalutamide therapy (CTC+/AR-FL+ cohort). [file 40364_2023_481_MOESM6_ESM.docx]

Table S3: Univariate analyses of biomarkers for PFS and OS in 65 mCRPC-patients on abiraterone or enzalutamide therapy (overall cohort)

Abbreviations: abi: abiraterone; ALP: alkaline phosphatase; CI: confidence interval; CTC: circulating tumor cell; enza: enzalutamide; Hb: hemoglobin; HR: hazard ratio; LDH: lactate dehydrogenase; PSA: prostate specific antigen

|  | **Progression Free Survival** | | **Overall Survival** | |
| --- | --- | --- | --- | --- |
| **Variable** | **HR (95% CI)** | **p** | **HR (95% CI)** | **p** |
| **Gleason-Score ≥ 8**  **No**  **Yes** | 1 (reference)  0.926 (0.426-2.010) | 0.846 | 1 (reference)  0.980 (0.483-1.985) | 0.954 |
| **Prior docetaxel**  **No**  **Yes** | 1 (reference)  1.077 (0.570-2.035) | 0.820 | 1 (reference)  1.693 (0.926-3.097) | 0.087 |
| **Prior abi or enza**  **No**  **Yes** | 1 (reference)  3.742 (1.892-7.401) | **<0.001** | 1 (reference)  3.772 (2.051-6.937) | **<0.001** |
| **Bone metastases**  **No**  **Yes** | 1 (reference)  1.937 (0.885-4.239) | 0.098 | 1 (reference)  2.337 (1.082-5.046) | **0.031** |
| **Visceral metastases**  **No**  **Yes** | 1 (reference)  1.441 (0.567-3.664) | 0.443 | 1 (reference)  2.295 (0.803-6.554) | 0.121 |
| **LDH elevated at baseline**  **No**  **Yes** | 1 (reference)  1.388 (0.766-2.517) | 0.280 | 1 (reference)  1.472 (0.836-2.590) | 0.180 |
| **ALP elevated at baseline**  **No**  **Yes** | 1 (reference)  1.806 (0.986-3.310) | 0.056 | 1 (reference)  2.294 (1.259-4.180) | **0.007** |
| **Hb** $\boldsymbol{\leq}$ **12 at baseline**  **No**  **Yes** | 1 (reference)  2.442 (1.198-4.977) | **0.014** | 1 (reference)  6.893 (3.406-13.949) | **<0.001** |
| **PSA doubling time < 3 months**  **No**  **Yes** | 1 (reference)  1.808 (0.993-3.292) | 0.053 | 1 (reference)  1.475 (0.833-2.611) | 0.183 |
| **Absence of PSA decline** $\boldsymbol{\geq}$ **50%**  **No**  **Yes** | 1 (reference)  0.254 (0.131-0.493) | **<0.001** | 1 (reference)  0.444 (0.249-0.791) | **0.006** |
| **CTC positivity**  **No**  **Yes** | 1 (reference)  3.966 (1.517-10.370) | **0.005** | 1 (reference)  5.243 (1.856-14.812) | **0.002** |

Table S4: Univariate analyses of biomarkers for PFS and OS in 54 mCRPC-patients on abiraterone or enzalutamide therapy (CTC+/AR-FL+ cohort)

Abbreviations: abi: abiraterone; ALP: alkaline phosphatase; AR-FL: androgen receptor full length; AR-V: androgen receptor splice variant; CI: confidence interval; CTC: circulating tumor cell; enza: enzalutamide; Hb: hemoglobin; HR: hazard ratio; LDH: lactate dehydrogenase; PSA: prostate specific antigen

|  | **Progression Free Survival** | | **Overall Survival** | |
| --- | --- | --- | --- | --- |
| **Variable** | **HR (95% CI)** | **p** | **HR (95% CI)** | **p** |
| **Gleason-Score ≥ 8**  **No**  **Yes** | 1 (reference)  0.624 (0.248-1.570) | 0.318 | 1 (reference)  0.659(0.297-1.460) | 0.304 |
| **Prior docetaxel**  **No**  **Yes** | 1 (reference)  1.380 (0.702-2.714) | 0.350 | 1 (reference)  3.128 (1.530-6.396) | **0.002** |
| **Prior abi or enza**  **No**  **Yes** | 1 (reference)  2.653 (1.333-5.278) | **0.005** | 1 (reference)  2.522 (1.349-4.715) | **0.004** |
| **Bone metastases**  **No**  **Yes** | 1 (reference)  0.576 (0.222-1.493) | 0.256 | 1 (reference)  0.958 (0.403-2.276) | 0.922 |
| **Visceral metastases**  **No**  **Yes** | 1 (reference)  1.728 (0.603-4.947) | 0.308 | 1 (reference)  2.898 (0.838-10.015) | 0.093 |
| **LDH elevated at baseline**  **No**  **Yes** | 1 (reference)  1.322 (0.701-2.494) | 0.388 | 1 (reference)  1.660 (0.908-3.037) | 0.100 |
| **ALP elevated at baseline**  **No**  **Yes** | 1 (reference)  1.259 (0.667-2.376) | 0.477 | 1 (reference)  1.665 (0.897-3.090) | 0.106 |
| **Hb** $\boldsymbol{\leq}$ **12 at baseline**  **No**  **Yes** | 1 (reference)  1.694 (0.823-3.488) | 0.152 | 1 (reference)  5.144 (2.500-10.581) | **<0.001** |
| **PSA doubling time < 3 months**  **No**  **Yes** | 1 (reference)  2.001 (1.044-3.837) | **0.037** | 1 (reference)  2.037 (1.065-3.896) | **0.032** |
| **Absence of a PSA decline** $\boldsymbol{\geq}$ **50%**  **No**  **Yes** | 1 (reference)  0.355 (0.180-0.700) | **0.003** | 1 (reference)  0.633 (0.348-1.151) | 0.134 |
| **AR-V positivity**  **No**  **Yes** | 1 (reference)  2.052 (1.013-4.160) | **0.046** | 1 (reference)  2.093 (1.085-4.039) | **0.028** |

Table S5: Univariate analyses of biomarkers for PFS and OS in 35 mCRPC-patients on abiraterone or enzalutamide therapy (CTC+/AR-FL+/AR-V+ cohort)

Abbreviations: AR-FL: androgen receptor full length; AR-V: androgen receptor splice variant; CI: confidence interval; CTC: circulating tumor cell; HR: hazard ratio

|  | **Progression Free Survival** | | **Overall Survival** | |
| --- | --- | --- | --- | --- |
| **Variable** | **HR (95% CI)** | **p** | **HR (95% CI)** | **p** |
| **1 AR-V**  **No**  **Yes** | 1 (reference)  0.901 (0.363-2.234) | 0.821 | 1 (reference)  0.528 (0.198-1.411) | 0.203 |
| **2 AR-Vs**  **No**  **Yes** | 1 (reference)  0.998 (0.462-2.158) | 0.997 | 1 (reference)  0.777 (0.368-1.641) | 0.509 |
| **3 AR-Vs**  **No**  **Yes** | 1 (reference)  1.086 (0.496-2.380) | 0.837 | 1 (reference)  2.063 (0.985-4.323) | 0.055 |

Table S6: Multivariate analyses of biomarkers for PFS and OS in 65 mCRPC-patients on abiraterone or enzalutamide therapy (overall cohort)

Abbreviations: abi: abiraterone; AR-FL: androgen receptor full length; AR-V: androgen receptor splice variant; CI: confidence interval; CTC: circulating tumor cell; enza: enzalutamide; Hb: hemoglobin; HR: hazard ratio; PSA: prostate specific antigen

|  | **Progression Free Survival** | | **Overall Survival** | |
| --- | --- | --- | --- | --- |
| **Variable** | **HR (95% CI)** | **p** | **HR (95% CI)** | **p** |
| **CTC positivity**  **No**  **Yes** | 1 (reference)  3.969 (1.386-11.367) | **0.010** | 1 (reference)  2.891 (1.0692-7.817) | **0.036** |
| **Prior abi or enza**  **No**  **Yes** | 1 (reference)  1.962 (0.947-4.066) | 0.070 | 1 (reference)  1.501 (0.675 -3.340) | 0.319 |
| **Absence of a PSA decline** $\boldsymbol{\geq}$ **50%**  **No**  **Yes** | 1 (reference)  0.835 (0.428-1.629) | 0.597 | 1 (reference)  0.358 (0.167-0.770) | **0.009** |
| **Hb ≤ 12 at baseline**  **No**  **Yes** | 1 (reference)  4.805 (2.336-9.882) | **<0.001** | 1 (reference)  1.807 (0.8543-3.822) | 0.122 |

Table S7: Multivariate analyses of biomarkers for PFS and OS in 54 mCRPC-patients on abiraterone or enzalutamide therapy (CTC+/AR-FL+ cohort)

Abbreviations: abi: abiraterone; AR-FL: androgen receptor full length; AR-V: androgen receptor splice variant; CI: confidence interval; CTC: circulating tumor cell; enza: enzalutamide; Hb: hemoglobin; HR: hazard ratio; PSA: prostate specific antigen

|  | **Progression Free Survival** | | **Overall Survival** | |
| --- | --- | --- | --- | --- |
| **Variable** | **HR (95% CI)** | **p** | **HR (95% CI)** | **p** |
| **AR-V positivity**  **No**  **Yes** | 1 (reference)  1.628 (0.784-3.383) | 0.191 | 1 (reference)  1.776 (0.894-3.539) | 0.101 |
| **Prior abi or enza**  **No**  **Yes** | 1 (reference)  1.628 (0.730-3.630) | 0.233 | 1 (reference)  1.947 (0.998-3.798) | 0.051 |
| **Absence of a PSA decline** $\boldsymbol{\geq}$ **50%**  **No**  **Yes** | 1 (reference)  0.504 (0.225-1.127) | 0.095 |  |  |
| **Hb ≤ 12 at baseline**  **No**  **Yes** |  |  | 1 (reference)  3.925 (1.859-8.286) | **<0.001** |
